# Indonesian registry on atrial fibrillation (OneAF)

**DOI:** 10.1097/MD.0000000000025725

**Published:** 2021-05-14

**Authors:** Sunu Budhi Raharjo, Agung Fabian Chandranegara, Dicky Armein Hanafy, Muhammad Yamin, Hauda El Rasyid, Haryadi, Ardian Rizal, Pipin Ardhianto, Dony Yugo Hermanto, Yoga Yuniadi

**Affiliations:** a Department of Cardiology and Vascular Medicine, Faculty of Medicine, Universitas Indonesia, National Cardiovascular Center Harapan Kita; b Pasar Rebo General Hospital; c Department of Cardiology and Vascular Medicine, Faculty of Medicine, Universitas Indonesia, National Cardiovascular Center Harapan Kita; d Department of Internal Medicine Faculty of Medicine, Universitas Indonesia, Cipto Mangunkusumo National General Hospital, Jakarta; e Department of Cardiology, Faculty of Medicine, Andalas University, M Djamil General Hospital, Padang, West Sumatra; f Eka Hospital, Pekanbaru, Riau; g Department of Cardiology, Faculty of Medicine, Brawijaya University, Syaiful Anwar General Hospital, Malang; h Department of Cardiology, Faculty of Medicine, Diponegoro University, Kariadi General Hospital, Semarang; i Department of Cardiology and Vascular Medicine, Faculty of Medicine, Universitas Indonesia, National Cardiovascular Center Harapan Kita, Jakarta, Indonesia.

**Keywords:** atrial fibrillation, hospital-based registry, Indonesia

## Abstract

**Background:**

Data on the optimal therapeutic international normalized ratio (INR) for non-valvular and valvular atrial fibrillation (AF) in Indonesia is currently unavailable. Therefore, we designed the Indonesian Registry on Atrial Fibrillation (OneAF) registry in order to seek a safe and beneficial range of INR in Indonesian patients with non-valvular and valvular AF.

**Methods/design:**

The OneAF registry is a nationwide collaboration of the Indonesian Heart Rhythm Society (InaHRS) enrolling all hospitals with cardiac electrophysiologists in Indonesia. It is a prospective, multicentre, nationwide, observational study aiming to recruit non-valvular and valvular AF patients in Indonesia. The registry was started in January 2020 with a planned 2 years of recruitment. There are 2 respondents for this registry: non-cohort and cohort respondents. Non-cohort registry respondents are AF patients at hospitals who fulfill inclusion and exclusion criteria but did not consent for a 24 month follow up. Whereas patients who consented for a 24 month follow up were included as cohort registry respondents. Key data collected includes basic sociodemographic information, symptoms and signs, medical history, results of physical examination and laboratory test, details of diagnostics and treatment measures and events.

**Results:**

Currently, a total of 1568 respondents have been enrolled in the non-cohort registry, including 1065 respondents with non-valvular AF (67.8%) and 503 respondents with valvular AF (32.2%). We believe that the OneAF registry will provide insight into the regional variability of anticoagulant treatment for AF, the implementation of rhythm/rate control approaches, and the clinical outcomes concerning cardiocerebrovascular events.

**Trial registration:**

Registered at clinicaltrials.gov (NCT04222868).

## 1 Introduction

Atrial fibrillation (AF) is one of the most common cardiac arrhythmias and a well-known risk factor for severe stroke.^[1]^ Long-term oral anticoagulant (OAC) agents such as the vitamin K antagonists (VKA) are the mainstay of treatment, effectively reduces the risk of stroke by two-thirds.^[2]^ Warfarin has been used for more than 50 years for this purpose, however, its use is not without caveats.^[3]^ This agent's pharmacokinetics and pharmacodynamics’ unpredictable nature causes fluctuation in the serum levels, thus requiring routine international normalized ratio (INR) surveillance.

Currently, the American College of Cardiology/American Heart Association and European Society of Cardiology guideline recommends using oral anticoagulant agents in patients with CHA2DS2-VASc score of 2 or above. Several anticoagulants can be used, however, if a VKA/warfarin were to be used, a therapeutic INR range of 2.0 to 3.0 is recommended in patients with non-valvular atrial fibrillation to prevent the occurrence of stroke.^[4,5]^ However, studies in Japan showed a different treatment response profile. Yasaka et al showed that INR of 1.6 to 2.6 was the “sweet spot” for reducing ischemic stroke without excess hemorrhagic complications.^[6]^ A randomized controlled trial indicates that low-intensity warfarin (INR 1.5 to 2.1) is safer compared to conventional-intensity (INR 2.2 to 3.5) regimen. The authors acknowledged the nature of “partial protection” of warfarin at INR 1.66 to 1.99, in the terms that this range of INR effectively prevents a large infarct and poor outcome, even when ischemic stroke did occur.^[7,8]^ These results are different from the therapeutic INR goals endorsed by the American College of Cardiology/American Heart Association and European Society of Cardiology, indicating possible heterogeneity due to sociodemographic factors.

Variation secondary to sociodemographic factors mandates each region to conduct its study to find its own respective INR “sweet spot”. Indonesia has a sociodemographic profile unique to the other regions that have conducted such studies. Contributing further to the difference is the high prevalence of rheumatic heart disease and valvular AF in Indonesia, unlike developed countries.^[9]^ Indonesia currently ranks at 4th in the largest populations of rheumatic heart disease patients, with approximately 1.18 million rheumatic heart disease cases in 2015.^[10]^ Approximately 30% of patients with untreated rheumatic heart diseases will develop AF, with mitral stenosis (29%) and mitral regurgitation (16%) being the most common culprit.^[9,11,12,13]^ Moreover, VKA is the only OAC that is covered by the National Health Insurance, highlighting the importance of VKA in Indonesia. This registry's rationale stems from the fact that due to the double-edged nature of VKA, the optimal INR range that may vary across different populations should be defined. This study intends to seek a safe and beneficial range of INR on Indonesian patients with non-valvular and valvular AF.

## 2 Methods

### 2.1 Study design/population

The Indonesian registry on atrial fibrillation (OneAF) is a prospective, multicentre, nationwide, observational study aiming to recruit AF patients aged ≥18 years in Indonesia. The registry was started in January 2020 with a planned 2 years of recruitment. We intend to follow-up the patients for 24 months. The OneAF registry is a nationwide collaboration of the Indonesian Heart Rhythm Society (InaHRS) and the Indonesian Ministry of Health, enrolling all hospitals with board-certified cardiac electrophysiologists in Indonesia. Currently, there are 16 hospitals involved in this project. We used a web-based data collection in which the data is submitted to the available online platform. The data gathered will be managed by the principal investigator and the assigned InaHRS team. Follow-up data will be obtained during hospital visits and by phone calls. InaHRS funded this study.

### 2.2 Ethical approval and patient consent for the study

This study adheres to the principles of the Declaration of Helsinki. Ethical clearance was granted by, and the information sheet and consent form approved by the Ministry of Health - Institutional Review Board. Written informed consent is obtained from all participants. In the case of participants who were blind or unable to read/write, verbal informed consent was obtained by a trained physician with the approval from an accompanying family member when available. Patient consent is obtained using forms. Explanatory forms regarding the purpose of consent were also distributed to patients. Explanatory forms are made using the format suggested by the Indonesian National Medical Research Ethical guideline. Physicians also give explanations to patients and next of kin regarding the purpose of consent. Patients are encouraged to ask in case they would like to know more. After explanations are given and the patient understands feedback, signatures are obtained on a consent form. A copy of the consent and explanatory form are given to the patient. Patients’ decision to participate in this study is not coerced or incentivized, nor will it alter their medical care at their respective hospitals. Patients can retract their consent anytime during their participation in this registry.

### 2.3 Study population and follow-up

All AF patients in the affiliated hospitals that fulfilled inclusion and exclusion criteria will be classified as respondents to the registry. The inclusion criteria for this study were:

1. Age >18 years old
2. Diagnosis of non-valvular and valvular AF.

While the exclusion criteria were:

1. patients with reversible AF
2. patients who have been enrolled as a respondent on other clinical studies.

**Figure 1:**
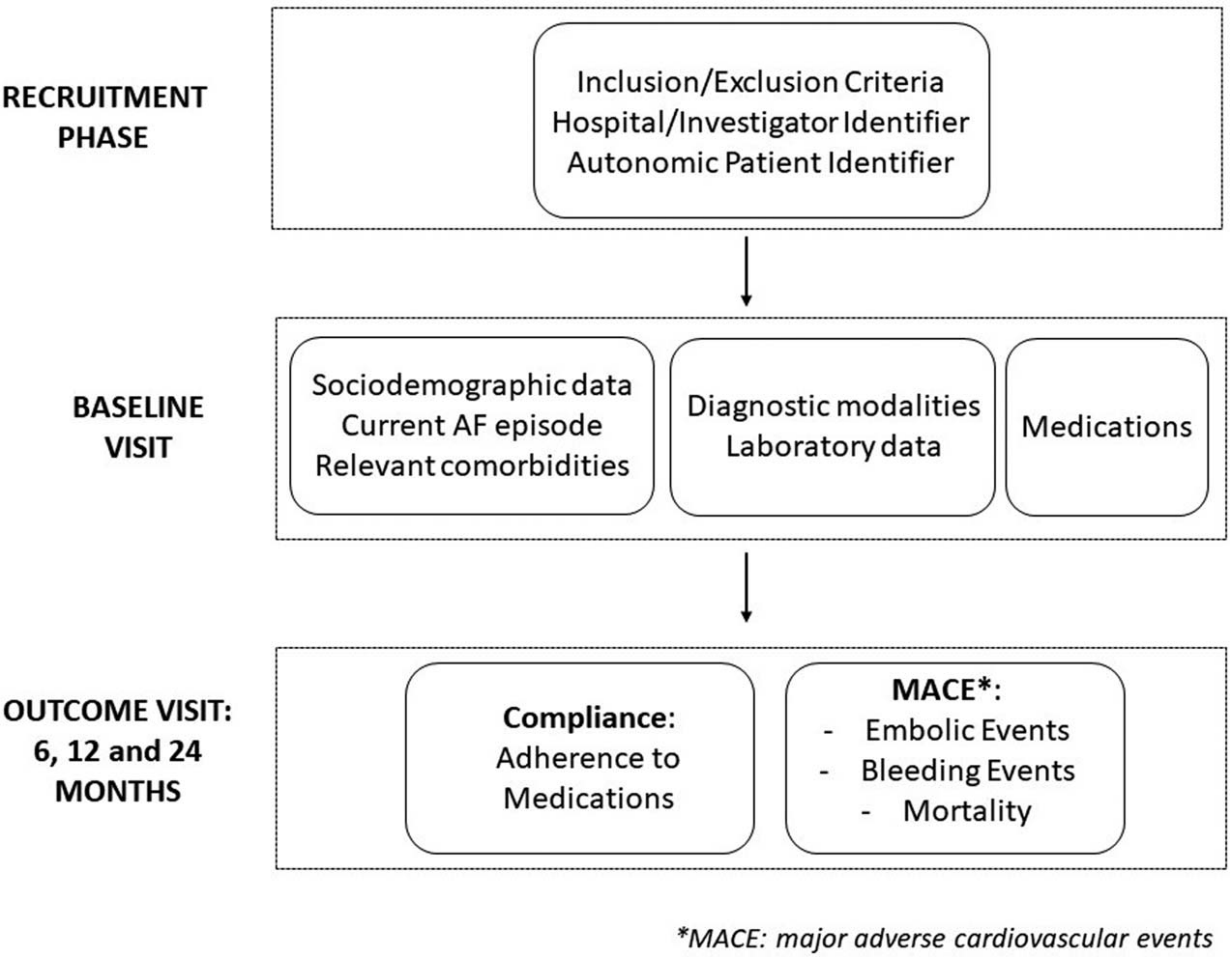
Project Structure.

The structure of this project is shown in Figure 1.

There are 2 respondents for this registry: non-cohort and cohort subjects. Non-cohort registry respondents are AF patients at hospitals who fulfilled inclusion and exclusion criteria but did not consent for a 24 month follow up. Baseline characteristics of these respondents will be put to eCRF according to their hospital medical records. The screening is performed to obtain:

1. demography and current episode,
2. history of atrial fibrillation and other comorbidities,
3. diagnostic studies and laboratory workups, and
4. medications (Table 1).

**Table 1:** Overview of baseline and outcomes variables.

Atrial fibrillation Data	History of AF, AF type/subtypes (non valvular/valvular) and type of valve (rheumatic mitral stenosis, regurgitation, prosthetic valve, post mitral valve repair), AF classification (paroxysmal, persistent, long-standing, permanent), EHRA Classification, CHADSVASC and HASBLED Score.• CHADVASC score were generated by collecting data regarding hypertension, diabetes mellitus, CHF, erosive gastritis, Stroke/TIA/systemic embolization, vascular disease• HASBLED Score were generated by collecting data regarding, history of uncontrolled hypertension, abnormal renal function, abnormal liver function, history of stroke, bleeding, labile INR, consumption of alcohol, presence of antiplatelet/NSAIDS medications.
Comorbid Data	History of cardiomyopathy, COPD, smoking, OSAS, thyroid disturbances, and hospitalization
Procedural History	History of AF Ablation, LAA surgery, MAZE procedure, ablate and pace, DC cardioversion, and date of each procedure
Diagnostic/Laboratory Result	Echocardiography, CT SCAN, PT, AST, ALT, serum creatinine, routine INR.
Medications	Subjects were inquired on the use of following agents:• Antiplatelet (ASA)• P2Y12 Inhibitor (Clopidogrel, prasugrel, ticagrelor, ticlopidine)• Oral VKA (warfarin, coumadin, phenprocoumon)• NOAC (apixaban, dabigatran, edoxaban, rivaroxaban)• Injectable anticoagulant (Fondaparinux, LMWH, UFH)• Heart rate related medications (beta blocker, digitalis, diltiazem, verapamil)• Rhythm control agents (amiodarone, disopyramide, dofetilide, dronedarone, flecainide, procainamide, propafenone, quinidine, sotalol)• Other drugs (ACE, ARB, statin)
Clinical Outcome	• Ischemic stroke (documented stroke or cerebrovascular accident caused by an ischemic event)• Peripheral embolism (abrupt vascular insufficiency associated with evidence of arterial occlusion in a vascular bed other than the cerebrovascular system)• All-cause mortality• Intracranial haemorrhages (bleeding into or around the brain)• Major bleeding (bleeding leads to hospitalization, transfusion, and/or involving a critial anatomic site)

Patients who fulfill inclusion and exclusion criteria and consent for a 24 month follow up were included as cohort registry respondents. Five milliliter venous blood sample will be collected from the cohort patients with consent. The sample will be centrifuged. Plasma and blood cells will be separately stored at −80°C refrigerators. The respondents will be followed up on the 6th, 12th, and 24th months. Follow-up data will be obtained during hospital visits and by phone calls. Follow up will be performed to collect information regarding:

1. events
2. AF and European Heart Rhythm Assocation (EHRA) classification
3. vital signs
4. history of any surgery/intervention
5. laboratory workup
6. change of medication
7. adherence to medication.

The observation will be ended on the 12th month or at the death of a patient.

The outcomes/events of interest are major adverse cardiovascular events, bleeding, and mortality. Major adverse cardiovascular events in this study is defined as the incidence of stroke/transient ischemic attack, thromboembolism, acute coronary syndrome. Bleeding events refer to intracranial bleeding and gastrointestinal bleeding. Mortality is defined as a clinically validated death.

### 2.4 Data collection and management

Baseline characteristics are obtained from the medical record. Follow-up data will be obtained on a subsequent visit to the heart clinic, otherwise, data will be obtained from a phone call. Data gathered will be put into eCRF. Data officers from each participating hospital will have access to eCRF for online data input. Username and password will be distributed to each data officer from the national registry coordinator to each participating hospital registry coordinator.

Baseline characteristics and several follow up data of registry subjects are obtained from the medical record. The recruitment of new subjects is performed in the first year. New patients/subjects of study will be interviewed for baseline characteristics. Baseline characteristics such as demographical data, current AF episode, history of arrhythmia, atrial fibrillation and other comorbidities, diagnostic workups, and medications. Follow-up data obtained from medical records consist of heart rate, blood pressure, EHRA classifications, history of intervention, and laboratory workups such as monthly INR, Prothrombin Time, serum creatinine, hemoglobin, and GFR. Other follow-up data will be obtained at revisiting to policlinic at the sixth and twelfth month since the first visit. Patients will be interviewed for events, medications, and a set of questions to assess medication adherence, this process lasts for approximately 10 minutes.

If a change in treating hospital occurs, the new hospital's data entry officer will perform an interview and follow up data entry. If the patients do not visit the OneAF registry participating hospital for follow up, data will be obtained via a phone call. Phone calls were done to obtain several data such as laboratory workup, vital signs, EHRA classifications, history of procedural interventions, adverse events experienced (if any), change of medications, and a set of questionnaires to assess medication adherence. Data will be input into eCRF by data entry officers from each hospital.

### 2.5 Statistical analysis

Statistical analysis will be performed by a team of certified biostatisticians using the Statistical Package on Social Sciences (SPSS) Software (IBM^TM^). Descriptive statistical analysis will be performed for baseline and follow-up variables. Categorical data is shown on bar charts with frequency and percentage. Continuous data will be shown with mean, minimum, and maximum. Univariate and multivariate analysis will be done for the outcomes to adjust findings to potential confounders. Subgroup analysis of valvular AF will be provided.

## 3 Results

### 3.1 Current status and data quality

**Figure 2:**
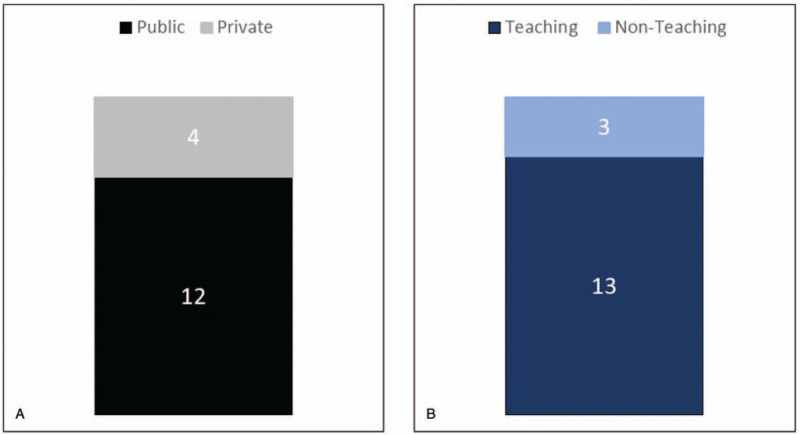
Characteristics of Participating Hospitals.

A total of 1568 respondents have been enrolled in the non-cohort registry, including 1065 respondents with non-valvular AF (67.8%), and 503 respondents with valvular AF (32.2%). The percentage of missing data was 18.7%, but there are no missing data among the mandatory variables which are controlled by the e-CRF system. These respondents were collected from 16 hospitals that have been involved in this project until now. The enlisted hospitals were distributed in several islands in Indonesia (Jawa, Sumatera, Sulawesi, Bali) and consisted of 12 public hospitals (75%) and 4 private hospitals (25%); 13 teaching hospitals (81.2%) and 3 non-teaching hospitals (18.8%) (Fig. 2).

## 4 Discussion

OneAF will serve as a ground for clinical decision making for AF patients, not only in Indonesia but also in the Southeast Asian populations. This registry will provide opportunities to answer various clinical questions such as patient's adherence to medication, efficacy and safety profile of VKA in Southeast Asian populations as compared to safety and efficacy profile osf existing data of east Asian and western patients.

The OneAF study is distinct from other large registries^[14–17]^ for several reasons. First, OneAF includes both non-valvular and valvular AF patients. The risk for stroke and bleeding in these 2 groups of patients are different. It will be interesting to observe how the patterns of therapies and the actual clinical outcomes of these 2 different populations. It should be noted that Indonesia ranks at 4th in the number of rheumatic heart disease in the world,^[10]^ therefore this registry will facilitate big data analysis in patients with valvular AF.

Secondly, this study involves both public and private hospitals, teaching and non-teaching hospitals in Indonesia. This will give better insights into the outcomes of different management strategies, including the use of oral anticoagulants. The majority of patients in public hospitals were anticoagulated using VKA (due to health care budget restrictions from the National Health Insurance), while patients in private hospitals were mostly anticoagulated using direct oral anticoagulants.

Thirdly, part of OneAF respondents will be followed-up longitudinally (cohort study) which enables evaluation of short and long terms effects of specific management. One of the most important clinical outcomes of AF is stroke, and unlike the other countries, stroke is the number 1 cause of mortality in Indonesia.^[18]^ Thus, data from this registry may potentially be helpful to reduce stroke-related mortality in Indonesia.

Genetic variation potentially cause alteration in response to anticoagulant, especially warfarin, and may contribute significantly to the clinical outcome.^[19–21]^ Asian populations generally has higher bleeding risk due to OAC, the risk of intracranial haemorrhage is approximately 4 times higher than that of Caucasians.^[22]^ This finding could not be explained solely by the lower time-to-therapeutic range in Asian compared to Caucasians, because Asian-Americans who achieved a median time-to-therapeutic rangesimilar to Caucasians have a significantly higher risk of intracranial haemorrhage.^[22]^ Interestingly, the bleeding rate was similar between Asian and Caucasian patients treated with a NOAC.^[23]^ This indicates that the Asians are more susceptible to bleeding during anticoagulant therapy, especially warfarin. Nevertheless, most of these studies were on East-Asian population. The OneAF registry is essential to delineate the role of genetic background in treating AF, particularly the Southeast Asian population, which is underrepresented.

## 5 Conclusion

The OneAF registry will provide insight into the regional variability of anticoagulant treatment for both non-valvular and valvular AF, the implementation of rhythm/rate control approaches, and the clinical outcomes concerning cardiocerebrovascular events. These observations will help provide information for decision making in the management of patients with non-valvular and valvular AF, especially in developing countries.

## Acknowledgment

The authors express gratitude to Raymond Pranata, Emir Yonas, Yuniar Shinta Dewi, Betania Ilhami for their excellent supports in preparing manuscript and collecting data.

## Author contributions

**Conceptualization:** Sunu Budhi Raharjo, Dicky Armein Hanafy, Yoga Yuniadi.

**Data curation:** Sunu Budhi Raharjo, Agung Fabian Chandranegara, Dicky Armein Hanafy, Muhammad Yamin, Hauda El Rasyid, Haryadi Haryadi, Ardian Rizal, Pipin Ardhianto, Dony Yugo Hermanto, Yoga Yuniadi.

**Formal analysis:** Sunu Budhi Raharjo.

**Funding acquisition:** Sunu Budhi Raharjo, Dicky Armein Hanafy.

**Investigation:** Sunu Budhi Raharjo, Agung Fabian Chandranegara, Dicky Armein Hanafy, Muhammad Yamin, Hauda El Rasyid, Haryadi Haryadi, Ardian Rizal, Pipin Ardhianto, Dony Yugo Hermanto, Yoga Yuniadi.

**Methodology:** Sunu Budhi Raharjo.

**Project administration:** Sunu Budhi Raharjo.

**Supervision:** Yoga Yuniadi.

**Validation:** Sunu Budhi Raharjo.

**Writing – original draft:** Sunu Budhi Raharjo, Agung Fabian Chandranegara, Dicky Armein Hanafy, Muhammad Yamin, Hauda El Rasyid, Haryadi Haryadi, Ardian Rizal, Pipin Ardhianto, Dony Yugo Hermanto.

**Writing – review & editing:** Sunu Budhi Raharjo, Yoga Yuniadi.
